# Long-Distance Three-Color Neuronal Tracing in Fixed Tissue Using NeuroVue Dyes

**DOI:** 10.1080/08820130701706711

**Published:** 2007-12-27

**Authors:** Heather Jensen-Smith, Brian Gray, Katharine Muirhead, Betsy Ohlsson-Wilhelm, Bernd Fritzsch

**Affiliations:** 1 Creighton University, Dept. of Biomedical Sciences, Omaha, Nebraska; 2 PTI Research, Inc., Exton, Pennsylvania, USA; 3 SciGro, Inc./Midwest Office, Madison, Wisconsin, USA; 4 SciGro, Inc./Northeast Office, Cambridge, Massachussetts, USA

**Keywords:** NeuroVue dyes, neuronal tract tracing, murine mutants, neuroembryology

## Abstract

Dissecting development of neuronal connections is critical for understanding neuronal function in both normal and diseased states. Charting the development of the multitude of connections is a monumental task, since a given neuron typically receives hundreds of convergent inputs from other neurons and provides divergent outputs for hundreds of other neurons. Although progress is being made utilizing various mutants and/or genetic constructs expressing fluorescent proteins like GFP, substantial work remains before a database documenting the development and final location of the neuronal pathways in an adult animal is completed. The vast majority of developing neurons cannot be specifically labeled with antibodies and making specific GFP-expressing constructs to tag each of them is an overwhelming task. Fortunately, fluorescent lipophilic dyes have emerged as very useful tools to systematically compare changes in neuronal networks between wild-type and mutant mice. These dyes diffuse laterally along nerve cell membranes in fixed preparations, allowing tracing of the position of a given neuron within the neuronal network in murine mutants fixed at various stages of development. Until recently, however, most evaluations have been limited to one, or at most, two color analyses. We have previously reported three color neuronal profiling using the novel lipophilic dyes NeuroVue (NV) Green, Red and Maroon (Fritzsch et al., Brain. Res. Bull. 66:249–258, 2005). Unfortunately such three color experiments have been limited by the fact that NV Green and its brighter successor, NV Emerald, both exhibit substantially decreased signal intensities when times greater than 48 hours at 37°C are required to achieve neuronal profile filling (unpublished observations). Here we describe a standardized test system developed to allow comparison of candidate dyes and its use to evaluate a series of 488 nm-excited green-emitting lipophilic dyes. The best of these, NV Jade, has spectral properties well matched to NV Red and NV Maroon, better solubility in DMF than DiO or DiA, improved thermostability compared with NV Emerald, and the ability to fill neuronal profiles at rates of 1 mm per day for periods of at least 5 days. Use of NV Jade in combination with NV Red and NV Maroon substantially improves the efficiency of connectional analysis in complex mutants and transgenic models where limited numbers of specimens are available.

## INTRODUCTION

A mechanistic understanding of how deficits contribute to nervous and sensory system disorders requires methods that can (a) delineate nerve fibers even in partially dysfunctional cells and (b) allow simultaneous tracing of multiple nerve tracts in embryos, juveniles and adults. Hodology, the study of connections between the nerve cells within the brain, is the most successful approach to establish the identity of neurons across phyla (Diaz et al., 2003; Fritzsch and Glover, 2006). During development, neurons use various cues to establish these connections (Kennedy et al., 2006). Mutating known or suspected cues and analyzing the resulting connectional deficits allows determination of the molecular basis for hodology (Gu et al., 2003). Neuroanatomy, the cornerstone of hodology, has moved from Golgi analysis in fixed tissue to vital and supravital dye tracing using a variety of actively transported dyes or dyes that diffuse within the cytoplasm (Kobbert et al., 2000; Lanciego et al., 2000). Methods have also been developed to detect co-localization of neuroanatomic probes (e.g., fluorescent dextran amines or lipophilic dyes) with molecular markers including immunocytochemical labels, GFP or radioactive in situ hybridization (Fritzsch and Hallbook, 1996; Kao and Sterling, 2003; Morris et al., 2006; Wouterlood et al., 2002).

Unfortunately, most connections are established before neurons are fully differentiated, limiting the ability to use immunocytochemistry for connectional analysis. In addition, most neurons can not be uniquely identified by phenotyping with antibodies. Even when antibodies exist that can be used reliably for identification in wild-type animals, they may not be useful in mutants because reduced synthesis of the epitope results in false negatives. To avoid such issues, it is paramount to have a robust and simple technique that is useful for labeling neurons at any stage and at any level of disruption of development and at any thickness of nerve fibers. Any neuron not in an advanced stage of apoptosis has cellular boundaries defined by a lipid bilayer, and labeling this lipid membrane is thus the most reliable way of defining neuroanatomic connections in normal and mutant development.

Lipophilic dyes provide unsurpassed single and dual color labeling of developing and postnatal tracts and neurons, and have therefore become an essential tool for dissecting both normal development and aberrant development caused by specific genetic manipulations (Fritzsch et al., 2005b). When studying complex models involving double, triple and/or conditional mutations, there is a strong need for multicolor analyses to best utilize rare and expensive-to-produce specimens. This requires sets of dyes that are (a) spectrally distinct but compatible with each other, (b) compatible with commonly used fluorescent proteins and immunohistochemical labels, (c) able to trace very thin (< 0.5 μm) fibers for distances of several millimeters, and (d) suitable for use not only in embryonic and neonatal specimens but also in juvenile and adult specimens, where anatomical distances to be traced are greater and increased signal-to-noise ratios are required due to myelin autofluorescence. We report here on the characterization of a new 488-nm excited lipophilic dye (NV Jade) that provides substantially improved detection distances compared with NV Green or NV Emerald and that can be readily imaged in combination with NV Red and NV Maroon using either epifluorescence or confocal microscopy.

## MATERIALS AND METHODS

### 

#### Lipophilic Dyes

NV Red, NV Maroon, NV Emerald, PTIR326, PTIR327 and PTIR330 (now NV Jade) were obtained from PTI Research, Inc. (Exton, PA). DiA and DiO were obtained from Invitrogen/Molecular Probes (Eugene, OR). Spectral characteristics for the different dyes are summarized in Table 1. NeuroVue dye-coated filters for neurotracing are commercially available through Molecular Targeting Technologies, Inc. (West Chester, PA).

**Table 1:** Spectral properties of selected lipophilic neurotracing dyes.

Dye	Absorption maximum* (nm)	Extinction Coefficient (M^−1^cm^−1^)*	Extinction maximum (nm)**	Emission maximum**(nm)
Red/Far Red dyes				
NV Red	565	100,533±5729	567	588
NV Maroon	650	216,700±907	647	667
Green dyes				
DiA	492***	49,000±4000	492	608
DiO	485***	153,000±7000	485	500
NV Emerald	492	132,400−134,610	494	507
PTIR 326	490	75,800	492	520
PTIR 327	450	79,400	449	473
NV Jade	478	78,083±8467	478	508

* Determined in ethanol using a Jasco V530 UV-Vis spectrophotometer; extinction coefficients at absorption maxima as determined from the slope of a plot of absorbance *vs*. concentration using 5 dye concentrations in the range of 0.5–10 μM.

** Obtained for 0.5–1.0 μM ethanolic solutions of dye using a Horiba Fluoromax-3 spectrofluorometer.

*** From Certificate of Analysis provided by Molecular Probes/Invitrogen.

#### Preparation of Dye-Coated Filters and Determination of Loading Levels

Nylon filters coated with individual dyes were prepared by soaking pre-weighed nylon filters for 2 minutes in varying concentrations of dye dissolved in dimethylformamide (DMF) (range 0.04–0.3M). After air drying overnight, dye loading was determined by subtracting pre-coating filter weights from post-coating filter weights. Co-coated dye filters loaded with reference dye (NV Red) and test dye (NV Jade, PTIR 326 or PTIR 327) were prepared by soaking pieces of nylon filter in DMF solutions containing approximately equimolar concentrations of each dye (range: 0.04–0.1M). Amounts of each dye present were quantified by cutting a segment from the coated filter, determining its weight as percentage of total filter weight, and extracting with 10 mL of ethanol under sonication until all the color was removed. Extracts were further diluted to a standard volume of 50 mL with ethanol and this solution diluted further as necessary to obtain absorbances in the 0.5–1.0 range. Absorbances were measured at 565 nm (A_565_), the maximum for NV Red, and at 490 nm, 450 nm or 478 nm for PTIR 326, PTIR 327 or NV Jade, respectively. Since all 3 green dyes have negligible absorbance at 565 nm (data not shown), the amount of NV Red on each filter section was calculated directly from absorbance at 565 nm (C_NV Red_ = A_565_/1.01×10^5^ M^−1^cm^−1^) and total volume of extract. Amount of green dye present on the same filter segment was calculated in the same fashion as described for NV Red, after first correcting for absorbance due to NV Red at relevant green wavelengths.

Correction factors were calculated from C_NV Red_ and NV Red extinction coefficients of 17,500 M^−1^cm^−1^ at 490 nm (PTIR326) or 9,700 M^−1^cm^−1^ at 478 nm (NV Jade). No correction was needed for PTIR327 since NV Red absorbance is negligible at 450nm. Filter loading was expressed as nmol/mm^2^, assuming that area % for the filter segment was the same as its measured weight %. Evenness of coating was assessed visually and dyes or coating concentrations that gave streaks or blotches were eliminated from further testing. Physical coating stability was also assessed visually by looking for dye transfer from the filter to the surface of the glassine envelope in which it was stored. Dyes exhibiting visible transfer during storage, handling or shipping were eliminated from further testing.

#### Thermal Stability Testing of Candidate Green Dyes

Neutral-buffered formaldehyde (NBF) was prepared for use in thermal stability testing by diluting formaldehyde (10% ultrapure EM grade, methanol free; Polysciences, Inc., Warrington, PA) to 4% w/v in 1X phosphate-buffered saline (PBS). Small strips (∼5 mm × 10 mm) were cut from each dye-coated filter, weighed, individually placed into separate capped vials containing 5 mL of 4% NBF and incubated at temperatures ranging from 22–62°C for periods of 0–25 days. At each time point, 3 strips were removed from the incubator, formalin was decanted and the strips were individually extracted with 10 mL of ethanol under sonication until all the color was removed. Amount of dye present (nmoles/mg) was determined by measuring absorbance at the appropriate absorption maximum, calculating concentration of dye present, multiplying by total volume of extract, and dividing by weight of the filter strip. Results were expressed as mean±standard deviation (SD) of triplicate samples, and kinetics of dye loss/degradation estimated from plots of dye remaining (nmol/mg) vs. time.

#### Preparation of Fixed Tissues

Neutral-buffered paraformaldehyde (NBPF) was prepared for use in tissue fixation and incubation with neurotracing dyes by dissolving 4% w/v paraformaldehyde in 0.1 M phosphate buffer and adjusting the pH to 7.4. All breeding and euthanasia was conducted following procedures approved by the Creighton University IACUC (protocol # 0630.1). For this study we used E18.5/P0 mouse embryonic spinal cords or heads of wild-type or several mutant lines with or without expression of GFP. Heterozygotic mice of several genes were crossed (Atoh1, Pou4f3, Neurog1, PLP-eGFP, HoxA2) and litters were recovered around birth. Mothers were euthanized through injection of Avertin followed by cervical dislocation after all reflexes had ceased. Newborn pups were anesthesthetized with Avertin; embryos received anesthesia through the mothers' blood supply. Embryos and newborn animals were pinned down on Sylgard covered dishes and perfused with 4% NBPF through the left ventricle for 1 minute using a peristaltic pump with glass capillaries attached to the tubes. After perfusion, heads were severed at the level of the upper limbs and the spinal columns were isolated by cutting away the ribs, viscera limbs and the tail. Perfusion-fixed heads were also prepared from 3-week-old mice anesthetized using Avertin to assess the behavior of test dyes in near-adult nervous tissue. Heads and spinal column preparations were stored in 4% NBPF at 4°C until used (typically between 1 week and 1 year, occasionally longer).

#### Spinal Cord Labeling and Preparation for Imaging

To allow accurate comparison of diffusion distances among dyes, we chose to apply the dyes by insertion of filter strips into coronal sections of the spinal cord as previously described (Fritzsch et al., 2005). After dye application, spinal column preparations (typically 3 per condition) were placed in 4% NBPF in a capped vial and incubated at 37–60°C for 24, 48 or 96 hours to allow for lateral diffusion. This method has significant technical advantages. In particular, razorblade slivers in a microblade holder (Geuder, Heidelberg, Germany) can be used to reliably place a small transverse cut between 2 adjacent neural arches. In our hands, the use of dye-coated pins (Chen et al., 2006) caused greater problems with spinal cord compression than the transverse cut method. Compression and tearing lead to uptake of dye in non-target cells, reducing the amount of dye available to label target cells and the rate at which target neuronal profiles are filled. The transverse cut method also has the advantage of creating a slot into which small segments cut from dye-coated filter strips will readily slide and be held securely in place by the adjacent neural arches, providing good topographical reproducibility of the dye insertion site. Finally, the use of dye coated filters rather than dye solutions or crystals makes it easier to apply well defined and reproducible amounts of dye and identify optimized loading levels of different dyes that give well-matched detection distances, simplifying experimental protocols by allowing labeling to be initiated simultaneously with all dyes.

At each time point, spinal columns were removed from the oven and held at 4°C in 4% NBPF until preparation for imaging. Imaging was typically done after the longest diffusion time for a given time series so that all spinal cords from each study could be directly compared on the same day using identical settings on the confocal microscope. Spinal columns were pinned down in a Sylgard coated dish, the neural arches were severed bilaterally along the spinal cord using microscissors (Geuder, Heidelberg), and the spinal cord was lifted from the spinal column. The caudal and rostral pieces of the spinal cord were placed in 100% glycerol between two cover slips so that the anterior and posterior halves were consistently positioned in a straight line, facing each other at the insertion site incision. The spinal cords were then covered with a third coverslip, generating a sandwich to hold the spinal cord in place for imaging. Great care was taken to place the spinal cord with the dorsal aspect facing the top coverslip. Glycerol mounted specimens were generally imaged promptly (i.e., maximum holding time of 1 hour at room temperature) but, in the unusual event that imaging could not be completed within the one hour time window, could be stored at 4°C overnight to preserve image quality.

#### Confocal Imaging and Estimation of Maximum and Relative Detection Distances in Spinal Cord

All images were taken on a Zeiss LSM 510 confocal microscope using identical instrument settings (Table 2) for a given dye, although power output obviously varied for the different lasers used for excitation. Using the composite tile setting of the Zeiss LSM confocal software we obtained images covering the entire diffusion distance from the cut site to where the dye blended into the background. To enhance spectral separation we always took images consecutively, thereby using the same PMT for imaging all dyes. High resolution composite images of each spinal cord were obtained from sequential images collected and integrated using Zeiss LSM 510 META imaging software. Pixel values of 4000 represented saturation. Based on the consensus of two independent observers, pixel values of 1000 were selected as the lowest detectable dye intensity that could reliably be distinguished from tissue background.

**Table 2:** Confocal and epifluorescence optics used for imaging selected lipophilic neurotracing dyes.

	Confocal Optics			
		
Dye	Excitation (nm)	Dichroic	Emission Filter Set	Epifluorescence Filter Set
Red/Far Red Dyes				
NV Red	543	NFT 545	565–615 nm	Texas Red
NV Maroon	633	NFT 490	650–710 nm	Cy5
Green Dyes				
DiA	488	NFT 490	500–550 nm	Texas Red
DiO	488	HFT 488	500–550 nm	FITC
NV Emerald	488	HFT 488	500–550 nm	FITC
PTIR 326	488	NFT 490	500–550 nm	FITC
PTIR 327	458	HFT 458	480–520 nm	FITC
NV Jade	488	NFT 490	500–500 nm	FITC

Intensity profiles generated along the medial aspect of each spinal cord were used to determine values for maximal or relative detection distances. Each experimental condition was run in triplicate, allowing up to 6 measurements per specimen (3 on the anterior and 3 on posterior part of the spinal cord). Maximal detection distance (MDD) was defined as the distance from cut point used for filter insertion to the point at which signal intensity dropped to an average pixel value of 1000. For filters co-coated with reference dye (NV Red) and test dye, relative detection distance (RDD) was defined as the MDD(test)/MDD(NV Red) × 100. Specimens with RDD values judged to be outliers at the 1% significance level (Marascuillo, 1971) were dropped and not used for calculation of group means or standard errors for MDD or RDD. NV Red was chosen as the reference dye for two reasons. First, it was readily distinguished spectrally not only from the candidate green dyes but also from NV Maroon, with which it would be used for 3-color neurotracing studies. Second, our previous work showed that NV Red could be detected in the same spectral window as DiI, the prototypic neurotracing dye, but gave four-fold greater signal at equivalent concentrations (Fritzsch et al., 2005b).

### Epifluorescence Imaging with Standard Filters

Since not all laboratories working on transgenic or mutant mice have full access to a high end confocal system such as the Zeiss LSM 510, dyes were also imaged on a CCD camera (Coolsnap ES, Photometrics) in a conventional microscope (Nikon E800) using standard commercially available filters (e.g., FITC, Texas Red and Cy5 filters for NV Jade, NV Red and NV Maroon, respectively). Where bleed through was seen (e.g., in the Cy5 channel used for imaging NV Maroon when bright NV Red labeled fibers were present in the same region), electronic corrections were made in each color channel using Image Pro (Media Cybernetics, Silver Spring, MD) prior to combining them into an RGB image. Background correction settings used by Image Pro can be generated only if bleed-through is unidirectional (e.g., bright NV Red signal may give some bleed through in the Cy5 filter used to detect NV Maroon, but bright NV Maroon signal does not give bleed through in the Texas Red filter used to detect NV Red).

### Tests for Longer Distance Double and Triple Labeling with NV Jade, NV Red and NV Maroon

To test the utility for imaging longer fibers than those in embryonic/neo-natal spinal cord, we labeled 3 different regions of the brain, each of which provides major inputs to the cerebellum, in order to evaluate the degree to which these fibers could be segregated using the improved 3 dye combination. Filter segments coated with NV Jade, NV Red or NV Maroon were inserted into the pontine nuclei, cerebellar cortex and the restiform body, respectively. Myelinization levels and associated autofluorescence background are markedly increased in juvenile and adult tissues compared with embryonic/neona-tal tissue (Cajal, 1919). This poses a significant signal to noise problem for other green dyes such as DiO and DiA. Therefore, we also investigated the ability of NV Jade to allow detection of very thin fibers in the brains of near-adult (18 day old) mice. Following dye application and incubation for 5 days at 37°C in 4% NBF, specimens were prepared for imaging as either whole mounts or 100 μm vibratome sections as previously described (Fritzsch et al., 2005; Gray 2006).

## RESULTS

### Selection of Lipophilic Green Dyes for Testing in Murine Spinal Cord Assay

**Figure 1:**
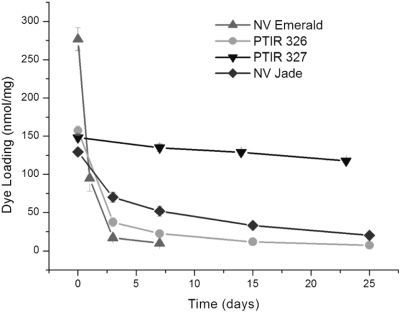
Thermal stability of selected green dyes at 37°C in 4% neutral buffered formaldehyde. Amount of intact dye remaining on dye-coated filter segments was determined for PTIR/NeuroVue dyes with excitation maxima near 488 nm as a function of incubation time and temperature (see Materials and Methods for details). The rank order of thermal stability was PTIR 327 best, followed by NV Jade, PTIR 326, and NV Emerald (see Table 3).

Dye diffusion distances in fixed tissue generally increase with incubation temperature, but exposure to elevated temperatures can also increase rate of degradation for some dyes, causing reduced detection distances and/or total loss of fluorescence as incubation times increase (Fritzsch and Gray, unpublished observations). We therefore pre-screened candidate dyes not only for their ability to coat filters with useable amounts of dye (assessed as solubility in DMF, the solvent used to prepare dye-coated filters, and the quality and physical stability of dye coating on nylon filters) but also for the thermal stability of dye-coated filter strips in neutral buffered formalin. As shown in Table 3 and Figure 1, all 3 green dyes tested (PTIR 326, PTIR 327 and NV Jade) had sufficiently good solubility, coating properties and improved thermal stability at 37°C to be considered as replacements for NV Emerald in many experiments. Therefore all three of these dyes were advanced to testing in the murine spinal Cord system using filters Co-coated with NV Red as a reference dye.

**Table 3:** Solubility, coating and thermal stability of selected lipophilic neurotracing dyes.

Dye	Solubility in DMF(mM)	Coating quality	Physical stability of dye coating	Thermal stability in 4% neutral-buffered formaldehyde at 37°C
Red/Far Red dyes				
NV Red	>150	Uniform	Stable to shipping	<5% loss@21 days
NV Maroon	>150	Uniform	Stable to shipping	∼25% loss@21 days
Green dyes				
DiA	∼50	Uniform	Stable to shipping	∼20% loss@21 days at 62°C
DiO	∼15	Uneven	Flakes off when handled	∼15% loss@21 days
NV Emerald	>140	Uniform	Stable to shipping	>50% loss@1 day
PTIR326	>100	Uniform	Stable to shipping	∼50% loss@2.5 days
PTIR327	>100	Uniform	Stable to shipping	<10% loss@23 days
NV Jade	>100	Uniform	Stable to shipping	∼50% loss@5.5 days

### Quantitation of Detection Distances in the Murine Spinal Cord Assay Using Co-coated Dyes

**Figure 2:**
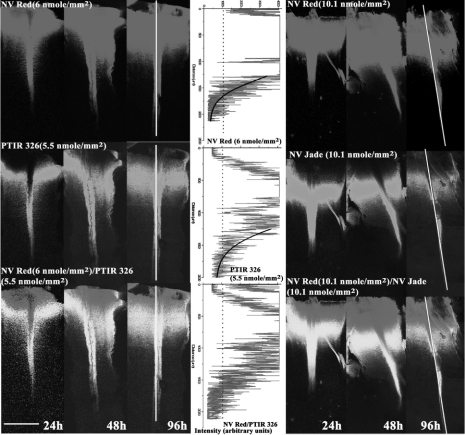
Determination of absolute and relative detection distances in murine spinal Cord assay. Nylon filters Co-coated with the indicated loadings of reference dye (NV Red) and test dye (PTIR 326 or NV Jade) were inserted in fixed E18.5/P0 murine spinal Cord preparations, incubated for the indicated time periods at 37°C in NBF, and imaged on the Zeiss LSM 510 Confocal microscope as described in Materials and Methods. Scale bar in lower left Corner indicates 500 μm. At each time point, intensity profiles (center panel) were generated along the medial aspect of each spinal Cord (shown as vertical white line in 96-h images), and maximum detection distance (MDD) for test and reference dyes was determined as the distance from the point of filter insertion to the point where average pixel intensities (black lines, Center panel) dropped to 1000 arbitrary units (consensus value between 2 independent observers for lowest intensity that Could reliably be distinguished from tissue background). Relative detection distance (RDD) was defined as RDD = MDD(test)/MDD(NV Red). MDD values for the images shown were as follows: NV Red/PTIR326:1150 μm/1250 μm, 1450 μm/1500 μm and 1675 μm/1750 μm at 24 h, 48 h and 96 h, respectively. NV Red/PTIR 330: 1300 μm/1450 μm, 1850 μm/1650 μm and 2000 μm/1800 μm at 24 h, 48 h and 96 h, respectively. Filter insertion point (see Materials and Methods) was used as the starting location for generation of intensity profiles because Combined images (bottom row) showed quenching of green dye emission by very high Concentrations of NV Red. This is seen as a red area (NV Red “only”) near the insertion point (top of each image) rather than the expected yellow (co-localized NV Red and green/test dye). Numbers of animals per group, group means and standard errors are listed in Table 3 for all time points.

The greatest detection distance for a given dye was typically seen in the fibers of the dorsal funiculus, the longest uninterrupted and most superficial tract in the spinal Cord (Figure 2). Since images for each reference/test dye pair were taken Consecutively using the same PMT (see Materials and Methods), they were always perfectly aligned, allowing direct measurement of maximal detection distance for each dye and relative detection distance for each test dye Compared to NV Red. Filters Co-loaded with varying but Comparable levels of test and reference dye (Table 4) were tested to allow determination of how dye Concentration affected both maximal and relative detection distances.

**Table 4:** Relative and absolute detection distances for selected lipophilic neurotracing dyes in E18.5/P0 murine spinal Cord.

Test Dye	Test Dye loading (nmole/mm^2^)	NV Red loading (nmole/mm^2^)	Time at 37°C	n	RDD, % (± SE)	Max. Detection Distance, Test Dye (mm±SE)	Max. Detection Distance, NV Red (mm±SE)
NV Maroon	8	8	24h	2	100 ± 0	1150 ± 100	1150 ± 100
			96h	2	100 ± 0	2838 ± 395	2838 ± 395
NV Jade	5.8	5.6	24h	4	94 ± 3.5	1633 ± 33	1683 ± 17
			48h	4	96 ± 5.5	2291 ± 110	2300 ± 50
			96h	3	92 ± 0.5	1916 ± 148	2075 ± 170
NV Jade	10.1	10.1	24h	4	104 ± 9.3	1375 ± 156	1366 ± 33
			48h	4	108 ± 2.3	1950 ± 126	1808 ± 102
			96h	4	106 ± 3.4	1875 ± 111	1775 ± 118
PTIR 326	5.5	6	24h	3	104 ± 4.8	1708 ± 262	1658 ± 287
			48h	6	104 ± 3.0	1725 ± 149	1658 ± 125
			96h	3	102 ± 1.6	1837 ± 114	1612 ± 185
PTIR 326	12.3	11.6	24h	5	106 ± 3.1	1254 ± 90	1170 ± 58
			48h	5	114 ± 4.9	2450 ± 93	2131 ± 62
			96h	4	95 ± 3.1	2125 ± 298	2237 ± 272
PTIR 327	34.7	24	24h	2	114 ± 14	1325 ± 100	1150 ± 100
			48h	3	104 ± 14	2025 ± 0	1675 ± 76
			96h	1*	0*	0*	2100*
DiA	5.6	6.1	24h	2	124 ± 6.6	1600 ± 100	1300 ± 150
			48h	2	107 ± 2.3	1950 ± 300	1825 ± 275
			96h	2	103 ± 0.5	2175 ± 75	2125 ± 125

* Representative specimen (one of 6 in 3 independent studies); others in group not measured.

One special Consideration when using Co-coated filters to determine relative detection distance is that high Concentrations of NV Red Can quench green dye emissions Close to the filter insertion site (tissue Cut point), due to significant NV Red absorption at 478 nm and 490 nm (see Materials and Methods). As Can be seen in Figure 2, such quenching would lead to an error in estimating relative diffusion distance if the site at which dye detection is first possible (the start site) rather than the Cut site were Chosen as the initial point from which to measure diffusion, since the start point is further down the spinal Cord than the Cut point. Maximal diffusion distance was therefore Calculated as the distance between the Cut site and the point at which mean intensity, determined using the intensity tool, fell below 1000 arbitrary units (Figure 2 and Materials and Methods). In this standardized spinal Cord model, both PTIR 326 and PTIR 330 gave detection distances similar to NV Red (Figure 2A, 2B; Table 4). In Contrast, even at very high filter loading levels (35X those tested for PTIR 326 and PTIR 330), PTIR 327 showed weaker initial signal to noise and became undetectable at 96 h (Table 4).

### Effect of Time and Filter Loading Levels on Detection Distance in the Murine Spinal Cord Assay

**Figure 3:**
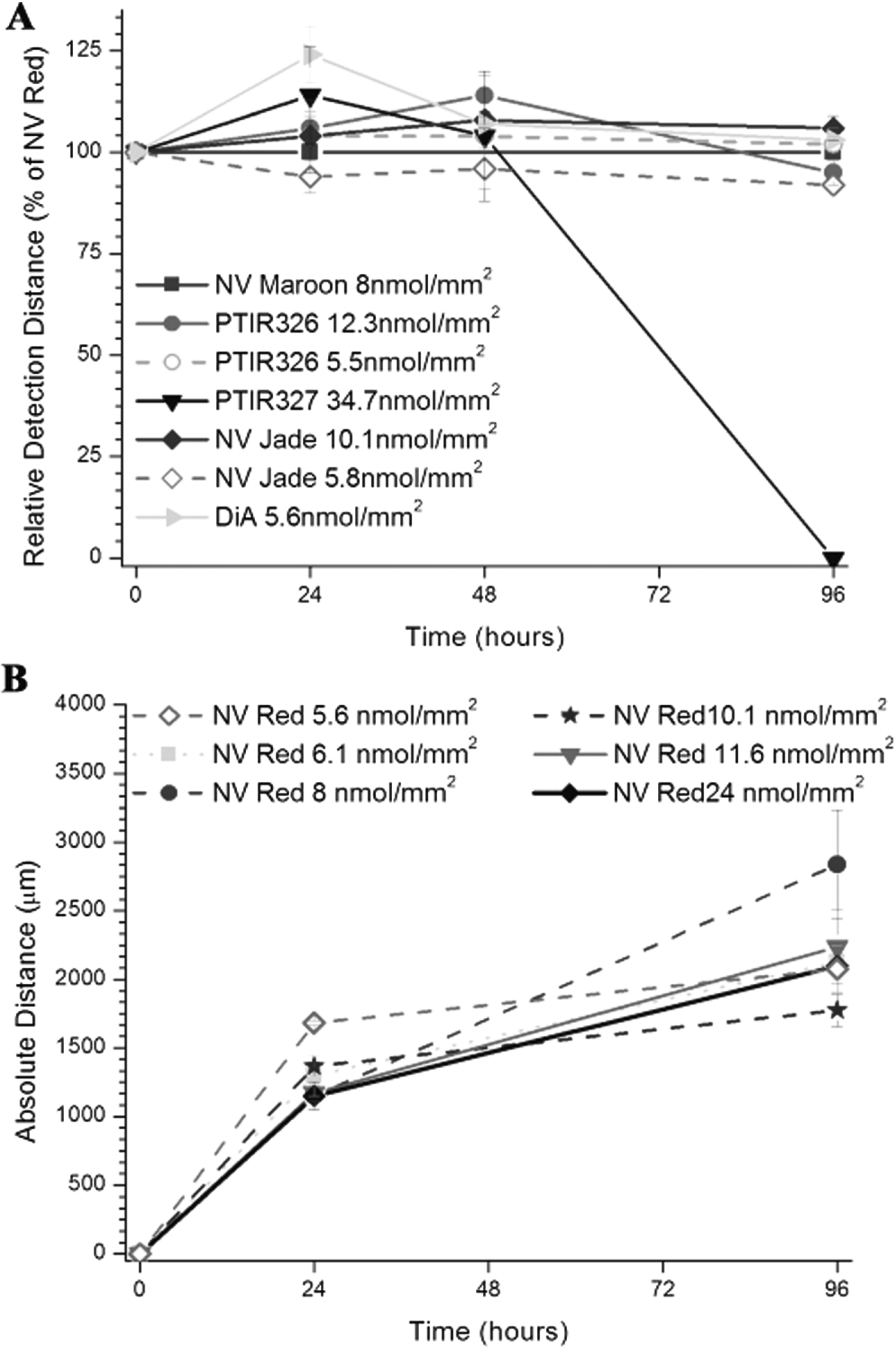

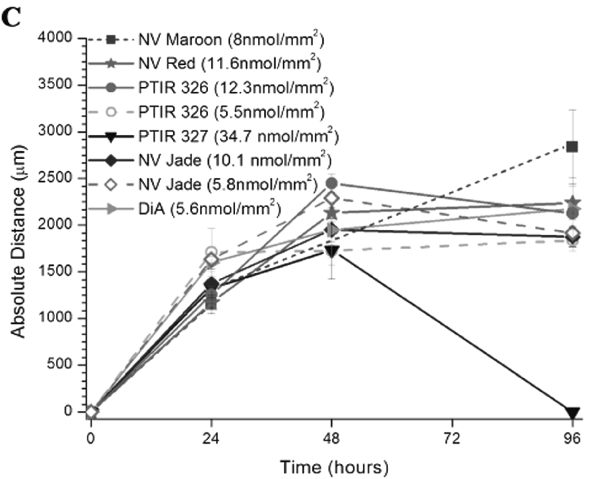
Relative and maximum detection distances for NV Maroon and Candidate green dyes in murine spinal Cord assay. Relative detection distance (RDD) and maximum detection distance (MDD) were Calculated as described in Figure 2 for nylon filters Co-loaded with the indicated levels of the listed dyes. Mean±SE is plotted for each time point, with the exception that only one of three samples was quantified at 96h for PTIR 327 (inverted triangle). Co-loading levels for NV Red and test dyes are shown in Table 4. **Panel A:** Within-replicate variability was significantly lower for RDD than MDD (Panels B and C) since ratiometric analysis reduces variability due to tissue-related factors such as differences in fixation time and Crosslinking. No significant differences in RDD values were observed across the different loading Concentrations for all tested dyes, including PTIR327, at 24 and 48h (F(6,15) = 1.83, p = 0.16, n.s.; F(6,17) = 0.76, p = 0.6, n.s.; respectively by single factor ANOVA). When PTIR327 was excluded, a marginally significant difference was noted for the remaining dyes (F(6,12) = 3.05, p = 0.047). However, it was attributable to an atypically low variance for the NV Jade triplicates at 5.8 nmol/mm^2^ loading and therefore must be Cautiously interpreted. **Panel B:** MDD values for NV Red increased at approximately 1 mm/day for the first 48h and then stabilized, presumably due to Complete filling of the relatively short neuronal profiles in neonatal spinal Cords. No dose-response effect was seen for different loading levels, which varied more than 4-fold.**Panel C**: MDD values for test dyes exhibited Comparable kinetics of profile filling to NV Red (again with the exception of PTIR 327) and were minimally affected by differences in loading level. No significant differences in MDD values were observed across the different loading Concentrations for all tested dyes at any time point.

The effect of loading levels on maximum and relative detection distances was determined as a function of time using filters Co-coated with NV Red and each test dye of interest. The latter included NV Maroon, PTIR 326, PTIR 327, NV Jade and DiA, a yellow-green dye previously demonstrated to be useful for long-distance tracing but suboptimal in terms of spectral overlap with NV Red (Fritzsch, unpublished observations). As shown in Figure 3A, RDD values in murine spinal Cord were found to be very Comparable for all dyes tested in the Co-coated filter assay (i.e., MDD values for test dyes were approximately 100% of MDDs for Comparable loading levels of NV Red when tested in the same tissue). This was true over a wide range of dye loading levels for both NV Red (5.6–24 nmol/mm^2^) (Figure 3B; Table 4) and test dyes (5.5–34.7 nmol/mm^2^) (Figure 3C; Table 4) again suggesting that there were no unexpected dye-dye interactions. With the exception of PTIR 327, which showed a dramatic decrease at 96 hours, RDD also remained essentially Constant over the 96-hour test period for all dyes (Figure 3A; Table 4). This apparent long term instability Combined with the much higher load needed to achieve diffusion Comparable to the Control dye and the inferior signal to noise ratio eliminated PTIR 327 from further testing.

Comparison of Figure 3A (relative detection distances referenced to NV Red) with Figure 3B and 3C (absolute detection distances for test dyes or NV Red, respectively) illustrates both the advantages and the limitations of using a ratiometric approach when evaluating the performance of Candidate neurotracing dyes. Specimen to specimen differences in tissue Crosslinking is expected to have similar effects on maximum detection distances for both test and reference dyes. Consistent with this expectation, expressing results as RDD (Figure 3A; Table 4) substantially reduces variability among replicates Compared with expressing them as absolute distances (Figure 3A, 3C; Table 4). This source of variability appeared to account for approximately half of total variation among replicates, which was not surprising given that fixation times for the specimens used in these studies varied from a few days to over 6 months.

Despite its significant advantages, expressing results as RDD does not allow assessment of the extent to which incubation time or dye loading affects maximal detection distance for a given dye. As shown in Figure 3B, maximum detection distance for NV Red increased rapidly for the first 48 hours, at a rate of approximately 1 mm/day. However, within experimental error, maximum detection distance at 96 hours was the same as at 48 hours, most likely because the limiting factor had become maximum neuronal fiber length in the embryonic/neonatal spinal Cord. For all of the other dyes tested except PTIR 327 (Figure 3C), essentially the same patterns were seen:, two-fold differences in dye loading had no significant impact on maximum detection distance (PTIR 326, NV Jade); maximum detection distances increased at ∼1 mm/day for the first 48 hours (all dyes); and little to no Change in maximum detection distance was seen from 48 to 96 hours (PTIR 326, NV Jade, NV Red, NV Maroon, DiA). In Contrast, despite the fact that PTIR 327-coated filter strips had better thermal stability in NBF than PTIR 326- or NV Jade-coated filter strips (Table 3), PTIR 327 fluorescence became undetectable between 48 and 96 hours (Figure 3C). It is not known whether this was due to greater losses due to transcellular diffusion, factors present in fixed tissue able to accelerate breakdown of this particular dye, or some other Cause. However, results in the spinal Cord model Clearly indicated that this dye was not suitable for use over the longer incubation times required for long-distance neurotracing in fixed tissue.

**Figure 4:**
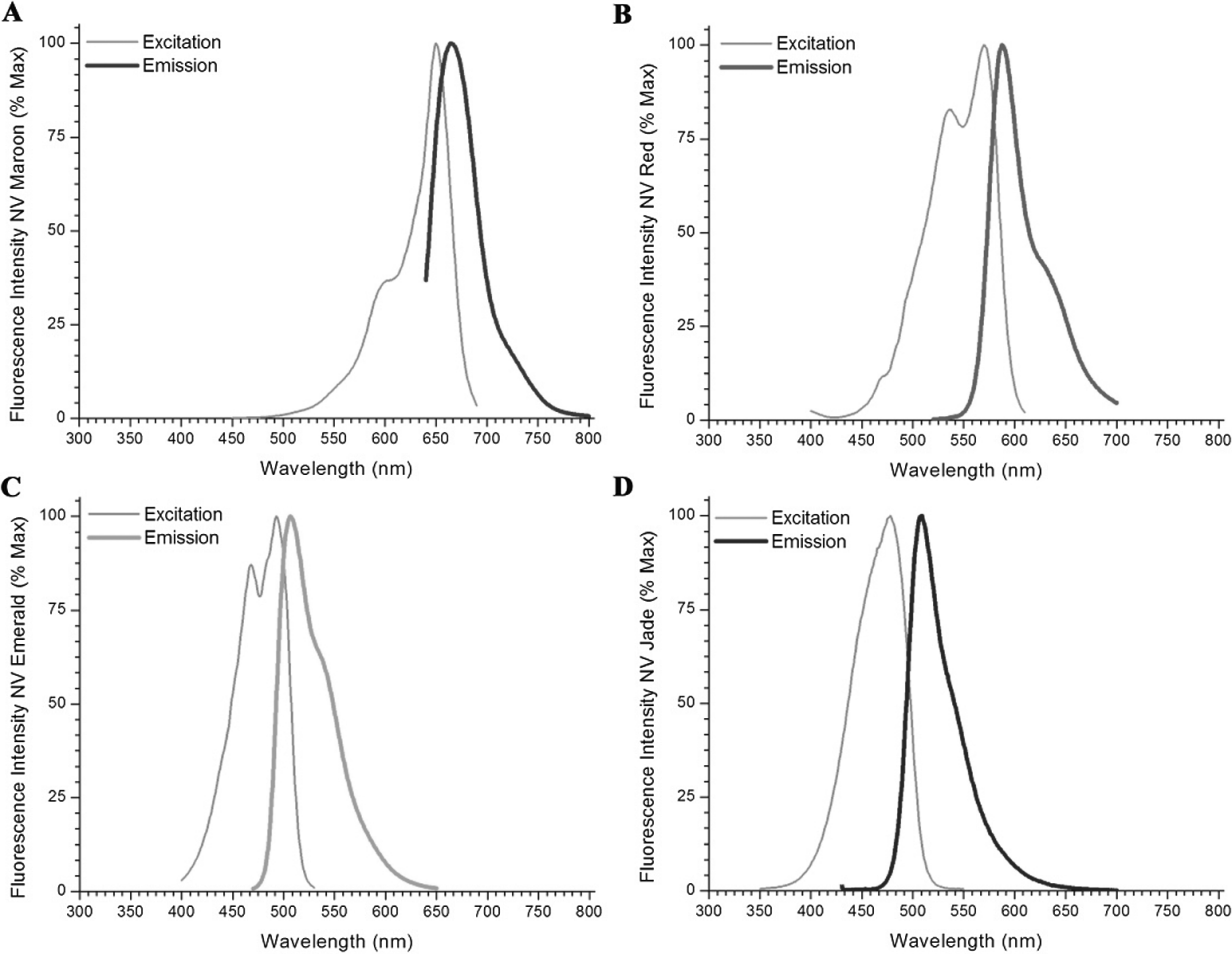
Excitation and emission spectra of NeuroVue Maroon, Red, Emerald and Jade.

As shown in Figure 3, both PTIR 326 and NV Jade gave RDD values (Figure 3A) and maximum detection distances (Figure 3C) in the spinal Cord model Comparable to those of NV Red and NV Maroon. However, because thermal stability was better for NV Jade than for PTIR 326 (Table 3), NV Jade was tentatively Chosen as the best 488-nm excited dye. As shown in Figure 4, NV Jade is similar to NV Emerald in its ability to be distinguished spectrally from NV Red and NV Maroon and is quiteompatible with standard optical filters used for confocal and epifluorescence imaging (Table 2). We therefore tested its utility for two and three color imaging in two additional murine systems where longer nerve tracts could be traced than in the neonatal spinal cord.

### Compatibility of NV Jade with NV Red and NV Maroon for Long-Distance 3-Color Neurotracing

**Figure 5:**
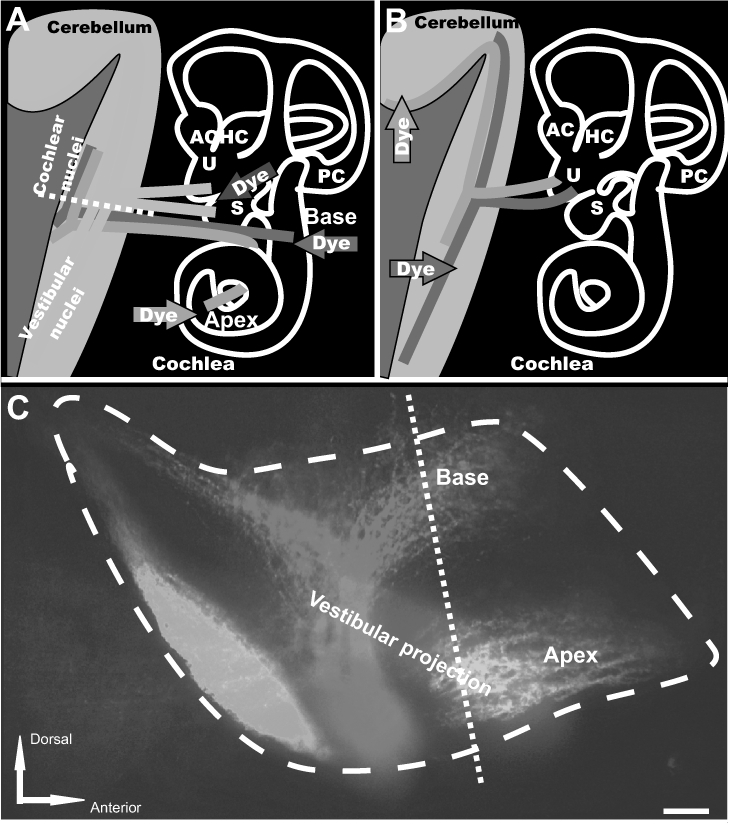

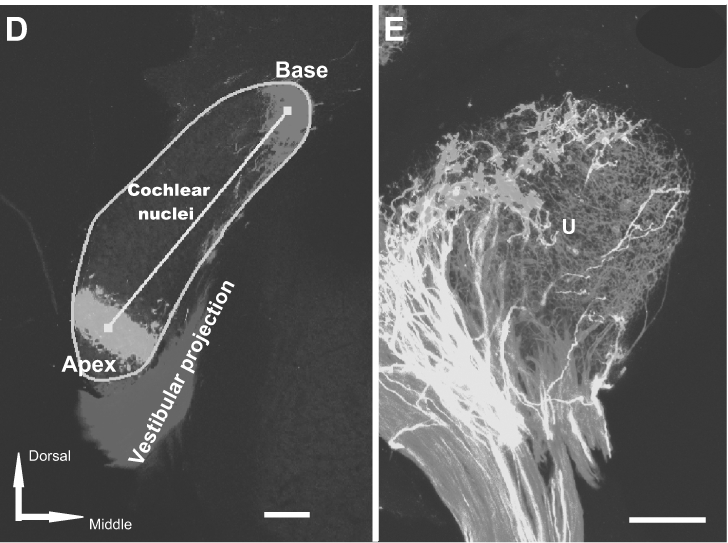
Three-color neurotracing with NV Jade, Red, and Maroon allows easy visualization of nerve fibers projecting from ear to brain or brain to ear by either epifluorescence or confocal microscopy. Nerve tracts originating at discrete locations in the periphery and projecting into closely adjacent regions in the brain of E18.5 mice (A, C, D) or nerve tracts arising in the brain and innervating the inner ear (B, E) were imaged using NV Jade (green pseudocolor) in combination with NV Red (red pseudocolor) and/or NV Maroon (blue pseudocolor) after 5 days of diffusion at 37°C Scale bar indicates 100 μm in all images. Abbreviations: AC, anterior crista; HC, horizontal crista; PC, posterior crista; S, saccule; U, utricle. **Panel A:** schematically illustrates the location of dye insertions in the inner ear (colored arrows) and the nerve fibers projecting from those locations back to the brain (colored lines), and in particular into the cochlear nuclei (red, green) and vestibular nuclei and cerebellum (blue). Epifluorescence of whole mounted brain and confocal images taken from tissue section shown by the white dotted line are shown in panel C (lateral view) and panel D (section along the dotted line shown in C), respectively. **Panel B:** shows insertion points in the brain (red and green arrows) used to fill nerve fibers projecting to the utricle (U), a vestibular endorgan in the inner ear. The corresponding confocal image is shown in panel E. **Panel C:** Nerve fibers originating from the base or apex of the cochlea or the central vestibule of the inner ear were labeled by insertion of filter segments coated with NV Jade, NV Red or NV Maroon, respectively. Epifluorescence imaging of a whole brain mount using standard FITC, Texas Red and Cy5 filters, respectively, showed excellent color segregation of the corresponding closely adjacent projection areas in the brain (cochlear nuclei and vestibular projection area). Image shown was corrected for bleed through in the Cy5 channel from regions having high intensity NV Red signal using Image Pro (see Materials and Methods). Dashed line indicates the level of the coronal section cut from the same preparation and imaged by confocal microscopy (panel D). Neuronal profile filling was detectable at distances of up to 5 mm away from the filter insertion sites. **Panel D:** A coronal 100 μm thick vibratome section cut along the dashed white line shown in panel A and imaged by confocal microscopy clearly shows the expected discrete projections from the inner ear into the cochlear and vestibular nuclei in the brain without any bleed-through. Using such projections, it is possible to determine the area of the cochlear nuclei (turquoise line), distances between projection bands (yellow line) or absence of overlap of specific projections in normal mice and assess how each is affected by specific mutations. **Panel E:** Insertion of NV Jade and NV Red filter segments into the cerebellum and the brainstem, respectively, of fixed neonatal mouse brain followed by confocal imaging of whole organ mount allows detailed visualization of segregation (green, red) or overlap (yellow) of innervating fibers within specific endorgans such as the utricle (U) of the inner ear, using a much simplified protocol compared to that require for neurotracing of the same tissue with DiA and DiI (see text).

**Figure 6:**
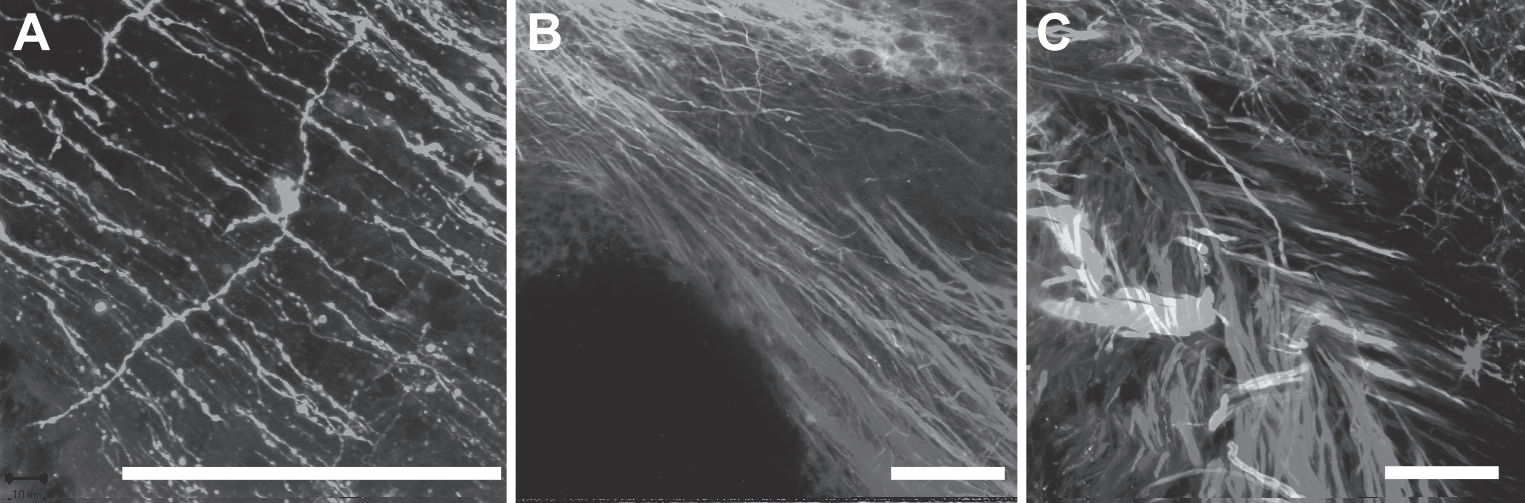
Three-color imaging with NV Jade, Red, and Maroon allows excellent resolution of thin fibers and discrete nerve tracts in near-adult murine brain. Nerve tracts projecting from one region of mature brain to another were labeled with NV Jade (green pseudocolor), NV Red (red pseudocolor) or NV Maroon (blue pseudocolor) and imaged by confocal microscopy after 5 days of diffusion at 37 C. Bar indicates 100 μm in all images. **Panel A:** Despite increased green autofluorescence due to myelinization of near-adult brain, NV Jade can be used in 18-day-old cerebella to label not only parallel fibers (green lines crossing from top left to bottom right) but also individual cells (stellar neuron in the center of A) at distances of up to 5 mm away from the injection site. (Representative image selected from 12 replicate specimens.) **Panels B and C:** After labeling in the pontine nuclei, cerebellar cortex and restiform body of the cerebellum with NV Jade, NV Red or NV Maroon, respectively, discrete fibers are consistently labeled and readily visualized near the deep cerebellar nuclei of P18 animals without bleed through and at distances of up to 1 cm away from the injection site over 50 animals. The same type of labeling was used on two replicate specimens, which were cut slightly differently to show fiber bundles running in parallel (B) or crossing one another (C).

Although the spinal cord model allows ready comparison of different dyes using a specimen that is relatively easy to prepare and image, the fibers of the dorsal funiculus from late embryonic stage and neonatal animals are shorter than many fibers of interest in the central and peripheral nervous systems of more mature animals. To evaluate the usefulness of the new 3-dye combination for long-distance tract tracing, we therefore investigated several longer and/or more difficult to label connections. We concentrated on the brainstem as this allowed us to label nerve tracts originating in the periphery and projecting into the brain or vice versa (Figure 5) or nerve tracts projecting from one region of the brain to another (Figure 6).

Panels C and D of Figure 5 illustrate the results obtained after 3 color labeling of nerve fibers arising from anatomically discrete areas in the inner ear and imaging in a region of the late embryonic mouse brain (E18.5) where the labeled fibers run closely adjacent to each other ("inner ear projection"). This projection is highly topographically organized, with fibers arising in the cochlea and projecting to the cochlear nuclei in the brain. Those central projections are adjacent to fibers arising in the vestibular part of the ear and projecting into the vestibular nuclei in the brain (Maklad and Fritzsch, 2003a). Inserting NV Jade, NV Red and NV Maroon loaded filter segments into the apex or base of the cochlea or into the vestibular region, respectively, and imaging their projections into the cochlear nuclei and vestibular nuclei, respectively, showed that excellent resolution among the 3 colors could be obtained using either epifluorescence imaging of a whole brain mount (Figure 5C) or confocal imaging of thick sections (Figure 5D).

The images shown in Figure 5C and Figure 5D represent normal brain architecture in a late embryonic stage mouse (E18.5). Figure 5E, on the other hand, shows fibers arising from two different regions in the brain of a newborn mouse that innervate the utricle, a balance-sensing endorgan in the inner ear. In this case, NV Jade and NV Red filter segments were inserted into the cerebellum and the brainstem, respectively, and confocal imaging was done using a whole organ mount (Gray, 2006). Previous studies, using a much more complex protocol requiring sequential labeling with DiI and DiA and different delay times for different developmental stages (Maklad and Fritzsch, 2002), had suggested that the cerebellar projecting fibers nearly exclusively innervate hair cells that are lateral to the striola part of the utricle. Using NV Jade and NV Red (Figure 5E), this could be clearly confirmed using a much simpler simultaneous labeling protocol.

Importantly, all of the images in Figure 5 were taken after 5-day incubations at 37°C and show excellent neuronal profile filling over distances of up to 5 mm away from the injection sites, confirming that NV Jade, NV Red and NV Maroon all continue to diffuse on average at rates of approximately 1 mm/day in these longer fibers. We therefore tested whether NV Jade allowed long distance filling and visualization of very thin unmyelinated nerve fibers in near-adult (P18) wild-type animals. Encouragingly, the multiple parallel fibers of the cerebellum, each in the order of 0.2–0.5 μm thick, were readily imaged after a 5-day incubation at 37°C at distances up to 5 mm away from the NV Jade insertion site in a different folium of the cerebellar cortex.

The degree of myelinization can easily be identified by the color (grey for unmyelinated areas, white for myelinated areas) under the dissection scope at the time of filter strip insertion. We also assessed the myelinated projections from 3 other regions of the brain (pontine nuclei, cerebellar cortex and restiform body) into the cerebellum by labeling each region with NV Jade, NV Red or NV Maroon, respectively. As shown in Figure 6, the resulting labeled fibers are readily imaged near the deep cerebellar nuclei of 18-day-old mice, again confirming that the improved 3-color neurotracing combination of NV Jade, NV Red and NV Maroon allows discrete fibers to be consistently labeled and fully segregated after 5 days of diffusion at 37°C and at distances of 5 mm and more from the dye insertion site(s) (Figure 6B, 6C). To further test the use of these dyes we have now evaluated their use in three different mutant lines in over 50 individuals and will be publishing those data soon.

## DISCUSSION

### 

#### Need for Multicolor Analysis

Neuroanatomy and neuroembryology have greatly benefited from technical advances allowing the study of single neurons and their interconnections. Most recently these include the ability to use molecular engineering to create sophisticated murine models with defined central or peripheral nervous system defects arising from single, multiple and/or conditional gene mutations (Beisel et al., 2005; Oury et al., 2006). Such models have the potential to define how altered gene expression affects the anatomy and physiology of nerve cell connections at the tissue, cell, subcellular and molecular levels. However, for this potential to be fully reached, the substantial time and cost required to produce complex mutants must be matched by methods allowing efficient characterization of their neurodevelopmental “phenotypes” in relatively small numbers of animals (ideally 6–10 per developmental stage).

Genetic alteration of a neuronal pathway frequently leads to early death of the organism, requiring that the altered gene be maintained as a heterozygote. For an early lethal phenotype, an average of 4 embryos is required to obtain a single animal bearing 2 copies of a single gene mutation. The yield of useful animals decreases, and the cost to produce each homozygous animal increases, as the number of mutated lethal genes desired for studying increases: 16 embryos are needed on average to obtain a single animal bearing two mutated copies of each of two damaged genes (4 × 4 animals of which a single one has the desired genotype if bred from doubly heterozygous animals), 64 embryos for triply homozygous animals (4 × 4 × 4 if bred from triply heterozygous animals), etc. Given an average litter size of 12 embryos, 5–6 litters are therefore needed to obtain each triply homozygous mutant animal to be studied.

These odds can be increased somewhat by using conditional mutants, especially if they are viable. For example, animals bearing doubly homozygous LoxP flanked genes can be bred with animals heterozygous for LoxP and single or multiple transgenes that express Cre, allowing conditional deletion of the LoxP flanked gene. This improves the odds to 1 embryo in 2–4 for single gene mutations, 1 in 8 for two gene mutations, 1 in 16 for three gene mutations, and 1 in 32 for 4 gene mutations, in theory enabling *in vivo* assessment of interactions among up to 4 genes. Unfortunately, the conditional approach is not always feasible, since widespread expression of genes used for the currently available Cre lines to eliminate the LoxP flanked gene(s) of choice often gives rise to adverse effects in organs not targeted (Ohyama and Groves, 2004). Robust, reliable multicolor techniques requiring only modest numbers of mutants for full assessment of neuronal phenotype at each developmental stage are therefore critical.

#### Current Neuronal Tracing Tools

A variety of methods has been used to study neuronal development and connectivity, but not all are well suited for efficient comparison of *in vivo* phenotypes in mutant *vs*. wildtype mice. Many mutations lead to incomplete differentiation of neurons or other cells that influence neuronal development. This can drastically reduce expression of key proteins or mRNAs (Fritzsch et al., 2005a, 2005c; Matei et al., 2005), leading to false negatives when using tools such as immunocytochemistry or in situ hybridization and to the conclusion that certain cell types or their connections are absent, whereas in fact the level of protein or mRNA has just fallen below the level of detectability. The systematic use of GFP, while extremely useful for the analysis of single gene mutations or wildtype brain development, actually slows down mutant analysis because GFP expressing transgenic lines (Lichtman and Conchello, 2005; Shaner et al., 2005) have to be crossed into the mutant lines, which then have to be bred back to homozygosity.

The preceding limitations can be avoided by using neurotracers that rely on intracytoplasmic movement along an axon, either by retrograde transport toward the neuronal cell body and dendritic tree, or by anterograde transport toward a synapse (Kobbert et al., 2000; Lanciego and Wouterlood, 2000; Reiner et al., 2000). Labeled species successfully used for this type of neurotracing have included: (a) macromolecules such as horseradish peroxidase (Fritzsch, 1993) and low or high molecular weight dextran amines (Fritzsch, 1993; Fritzsch and Sonntag, 1991; Fritzsch and Wilm, 1990; Glover et al., 1986; Nissen et al., 2005; Stirling et al., 1995); (b) micelles and/or membrane vesicles containing lipophilic dyes (Kobbert et al., 2000; Reiner et al., 2000); and (c) fluorescein-labeled microparticles (Chi et al., 2007; Kobbert et al., 2000). Such methods require viable cells, which means that they cannot be used in fixed tissues and for long-distance tracing in juveniles and adults, or require the presence of active transport mechanisms rather than simple passive diffusion. Another significant limitation for use of such tracers in multicolor color studies is that the staining protocols required to clearly distinguish the different tracers are relatively complex and time consuming (Lanciego 2000; Reiner 2000).

#### Lipophilic Dyes as Neuronal Tracing Tools

Lipophilic dyes have also proven highly useful for “post-mortem” tracing of neuronal connections in fixed tissue, due to their ability to intercalate into and diffuse laterally along the lipid bilayer of neuronal plasma membranes (Agmon et al., 1993; Chen et al., 2006; Fritzsch et al., 2005b; Godement et al., 1987; Hofmann and Bleckmann, 1999; Kao and Sterling, 2003; Lukas et al., 1998). The use of lipophilic dyes for multicolor neurotracing studies of the type needed to more efficiently analyze complex mutants has been very limited. In large part, this was due to the fact that the dyes most commonly used for double color studies had poorly matched spectral and diffusional properties and therefore required time consuming and cumbersome methodology to combine. However, our results with the green dyes studied here suggest that some of the apparent differences in diffusional performance reported in the literature may in fact have arisen from differences related to incubation temperatures for dyes with varying thermal stability.

The power of a multicolor approach is evident even using only two colors. For example, initial work using the relatively difficult 2-color DiA/DiI combination (Maklad and Fritzsch, 2002) has recently been extended using the better matched NV Red/NV Maroon combination (Fritzsch et al., 2005), revealing several features of inner ear-brain connectivity not detectable using single color techniques. These include the fact that the central projection to the cerebellum is partially segregated into linear and angular processing systems (Beisel et al., 2005; Maklad and Fritzsch, 2003b). Use of the NV Red/NV Maroon combination not only halved the number of mutants required but also revealed for the first time that delayed expression of brain-derived neurotrophic factor (BDNF) in the basal turn of the cochlea is crucial to avoid redirection of vestibular fibers into the cochlea (Tessarollo et al, 2004). Lipophilic dyes can be combined with gene expression detection systems such as GFP (Morris et al., 2006) and β-galactosidase (Karis et al., 2001) or with immunofluorescence targeted toward surface epitopes, provided the processing is done at low temperature (4°C) and without detergents to minimize lipophilic dye losses (Fritzsch, unpublished).

#### Three-Color Neuronal Tracing

Despite the obvious potential advantages of 3-color neurotracing, very few studies in the literature have used triple labeling to simultaneously identify a neuron and two of its inputs or nested sets of neurons that project to different areas (Fritzsch et al., 2005b; Gan et al., 2000; Hellmann and Fritzsch, 1996; Maklad and Fritzsch, 1999). We previously reported on the use of two lipophilic dyes that have equal or better neurotracing properties than existing dyes, NV Maroon and NV Red (Fritzsch et al., 2005b). Although these dyes provide a two color combination with well resolved spectra and well-matched diffusion properties, previously available green (488 nm excited) dyes used for lipophilic neurotracing have had significant limitations for use as a third color. DiO has good spectral compatibility and has been reported to work in some systems but not others (Honig, 1993; Honig and Hume, 1989; Wilm and Fritzsch, 1993), possibly due to its lower solubility.

DiA's relatively long Stokes' shift causes spectral overlap with DiI and NV Red. NV Green (Fritzsch et al., 2005b) had limited solubility and has been replaced by NV Emerald. NV Emerald exhibits good spectral compatibility (Figure 4) and excellent solubility in DMF (Table 2), allowing loading of the requisite amount of dye onto nylon filters, but exhibits thermal instability and loss of fluorescence with incubations longer than 48 hours at 37°C. (Figure 1). This makes it useful for 3-color tracing in embryonic and neonatal specimens but not for longer-distance tracing in juvenile and adult specimens, unless the species under study is one like fish, in which incubations can be carried out at lower temperatures.

#### NeuroVue Jade

The studies presented here describe work done in our laboratories to generate an improved green NeuroVue dye suitable for long-distance 3-color neurotracing studies. These include: (a) development of a standardized 2-color spinal cord assay system in which spectrally distinct test and reference dyes are co-coated on nylon filters to allow detection properties to be compared independent of extent of tissue cross-linking or other variables that can affect absolute diffusion distances in fixed tissue, and (b) development of a simple thermal stability test that can be used to rapidly eliminate dyes unstable to incubation at elevated temperatures in NBF, minimizing the number of animals and tissue specimens required. We suspect that differences in thermal stability may also explain some of the conflicting results in the literature for other green lipophilic tracers, wherein some studies reported successful labeling in systems requiring room temperature incubation (24°C), while others requiring incubation at higher temperatures (36–60°C) reported failure (Honig, 1993; Reiner et al., 2000). Precisely which chemical features define thermal instability remains unresolved at present, although in general the red and far red fluors appeared to have better stability than the green ones. Nonetheless, our simple thermal stability test (Figure 1, Table 3) helps to distinguish between more and less useful dyes.

All of the new green dyes tested in these studies (PTIR 326, PTIR 327 and NV Jade; Table 1) showed improved thermal stability compared to NV Emerald and comparable thermal stability to NV Red and NV Maroon (Table 3). Assessment in the spinal cord assay system (Figure 2) showed that all 3 of these dyes gave maximum detection distances that increased at the rate of approximately 1 mm/day for up to 48 hours when incubations were carried out at 37 C, a rate comparable to those for NV Red and NV Maroon in this system (Figure 3 and 4) and essentially independent of filter loading level in the range of 5.5–34.7 nmol/mm^2^ (Figure 3). For PTIR 327, fluorescence disappeared completely between 48 and 96 hours, indicating that although NV Red signal was stable some unknown factor not present in the thermal stability assay was causing loss/breakdown of this dye in fixed tissue specimens. For PTIR 326 and NV Jade, no further increases in maximum detection distance were noted between 48 and 96 hours, suggesting that maximum fiber length had been reached in this test system.

Direct comparison with performance of other 488 nm excitable lipophilic dyes shows that DiO has good thermal stability (Table 3). Unfortunately, it requires higher loading concentrations for equivalent detection distances (Fritzsch, unpublished observations) but cannot be loaded on to a filter at greater than 5–6 nmol/mm^2^ due to insufficient solubility and gives poor coating quality even at this relatively low level (Table 3). DiA has good solubility and coating quality in addition to good thermal stability (Table 3) and gives detection distances comparable to DiI, DiD, NV Jade, NV Red and NV Maroon (Fritzsch and Gray, unpublished observations). However, unlike NV Jade, DiA has a very large Stokes shift (Table 2), making its emission much more difficult to distinguish from DiI, NV Red and other dyes that excite near 560 nm unless they are imaged sequentially in a confocal microscope. If used with a confocal system, DiA could be a useful additional dye to be separated from the other dyes excitable near 488 nm, 568 nm and 645 nm. However, in normal epifluorescent imaging mode, DiA is difficult to distinguish from longer wavelength excitable dyes (Maklad and Fritzsch, 2003b), whereas NV Jade is much easier to segregate spectrally from such dyes (Figure 5).

NV Jade was therefore chosen as the best dye for evaluation in longer term studies tracing longer fibers based on its better thermal stability compared with PTIR 326 (Figure 1), its comparable detection distances at similar concentrations compared with NV Red and NV Maroon in the 4 day neonatal murine spinal cord assay (Figure 3), and its good spectral segregation from longer wavelength dyes (Figure 4). When combined with NV Red and/or NV Maroon, NV Jade was found to give excellent color segregation in 2-and 3-color neurotracing studies of overlapping/adjacent nerve fibers projecting into the brain from discrete anatomic locations in the inner ear (Figure 5C, 5D) or from discrete regions of the brain into a single end organ in the inner ear (Figure 5E), using either confocal or epifluorescence imaging. Additionally, NV Jade was shown to allow lipophilic neurotracing of thin fibers (0.2–0.5 μm thickness) in near-adult murine brain (Figure 6), a setting where autofluorescence due to myelinization becomes an increasing problem for DiO, DiA and other 488 nm excited dyes (B. Fritzsch, unpublished observations). In all of these systems, NV Jade was readily detected at locations 5 mm or more distant from the injection site after incubation for 5 days at 37°C. Waiting for longer times in near adult brain results in labeling of over 1 cm (the farthest we have done some preliminary tests in neonates).

Taken together, these data strongly support the conclusions that a) NV Jade is an improved green neurotracing dye that (a) for the first time allows long distance (> 5 mm), 3-color tract tracing in fixed tissue, and (b) its addition to the NeuroVue family will greatly facilitate efficient analysis of fixed nervous tissue from embryonic and postnatal murine mutants, using simple protocols in which labeling with all three dyes is initiated simultaneously. Finally, the work shown here has used examples drawn from neurodevelopment of the auditory system to illustrate the utility of this new 3-dye combination. However, we anticipate that it will prove broadly useful in many areas, including neuroimmunology, where there is strong evidence that immune cells and neuroimmune molecules such as cytokines, chemokines, and growth factors interact with critical neurodevelopmental processes and that exposure to neuroimmune challenges early in life may affect brain development.
